# Exploring Google Searches for Out-of-Clinic Medication Abortion in the United States During 2020: Infodemiology Approach Using Multiple Samples

**DOI:** 10.2196/33184

**Published:** 2022-05-12

**Authors:** Sylvia Guendelman, Elizabeth Pleasants, Coye Cheshire, Ashley Kong

**Affiliations:** 1 Wallace Center for Maternal, Child, and Adolescent Health School of Public Health University of California, Berkeley Berkeley, CA United States; 2 School of Information University of California, Berkeley Berkeley, CA United States; 3 Computing, Data Science, and Society Program University of California, Berkeley Berkeley, CA United States

**Keywords:** abortion, abortion access, internet, online information, Google searches, infodemiology

## Abstract

**Background:**

As access barriers to in-person abortion care increase due to legal restrictions and COVID-19–related disruptions, individuals may be turning to the internet for information and services on out-of-clinic medication abortions. Google searches allow us to explore timely population-level interest in this topic and assess its implications.

**Objective:**

We examined the extent to which people searched for out-of-clinic medication abortions in the United States in 2020 through 3 initial search terms: home abortion, self abortion, and buy abortion pill online.

**Methods:**

Using the Google Trends website, we estimated the relative search index (RSI)—a comparative measure of search popularity—for each initial search term and determined trends and its peak value between January 1, 2020, and January 1, 2021. RSI scores also helped to identify the 10 states where these searches were most popular. We developed a master list of top search queries for each of the initial search terms using the Google Trends application programming interface (API). We estimated the relative search volume (RSV)—the search volume of each query relative to other associated terms—for each of the top queries using the Google Health Trends API. We calculated average RSIs and RSVs from multiple samples to account for low-frequency data. Using the Custom Search API, we determined the top webpages presented to people searching for each of the initial search terms, contextualizing the information found when searching them on Google.

**Results:**

Searches for *home abortion* had average RSIs that were 3 times higher than self abortion and almost 4 times higher than buy abortion pill online. Interest in home abortion peaked in November 2020, during the third pandemic wave, at a time when providers could dispense medication abortion using telemedicine and by mail. *Home abortion* was most frequently queried by searching for *Planned Parenthood*, *abortion pill*, and *abortion clinic*, presumably denoting varying degrees of clinical support. Consistently lower search popularity for *self abortion* and *buy abortion pill online* reflect less population interest in mostly or completely self-managed out-of-clinic abortions. We observed the highest interest for home abortion and self abortion in states hostile to abortion, suggesting that state restrictions encourage these online searches. Top webpages provided limited evidence-based clinical content on self-management of abortions, and several antiabortion sites presented health-related disinformation.

**Conclusions:**

During the pandemic in the United States, there has been considerably more interest in home abortions than in minimally or nonclinically supported self-abortions. While our study was mainly descriptive, showing how infrequent abortion-related search data can be analyzed through multiple resampling, future studies should explore correlations between the keywords denoting interest in out-of-clinic abortion and abortion care measures and test models that allow for improved monitoring and surveillance of abortion concerns in our rapidly evolving policy context.

## Introduction

While abortion is a common pregnancy outcome that is currently legal in the United States, laws and policies across states pose substantial challenges to if, when, and how people can access abortions [1,2]. Restrictive laws and policies are multifold. They include gestational age limits, mandated counseling and waiting periods, parental involvement, public funding restrictions, and onerous requirements for abortion clinics and providers to operate and deliver services [2,3]. In addition, several states have imposed restrictions on the provision of abortion via telemedicine, disallowed the mailing of abortion medication to patients, and introduced trigger laws that would make abortion illegal if Roe v. Wade is overturned [2,4].

During the first year of the COVID-19 pandemic, while there was some protection of abortion services instituted in 13 states, additional challenges to access were introduced with states designating abortion as a “non-essential” or “elective” service [5], increased financial and administrative barriers for abortion clinics [6], and clinic closures [7]. Strong advocacy by proabortion groups helped to temporarily lift the national limitation on telemedicine abortion medication in the latter half of 2020 [8], and again in April of 2021 [9]. This has allowed patients to talk with a doctor by video or phone and then receive abortion pills in the mail to manage a home abortion if they live in a state allowing telemedicine abortion [9]. At the same time, people faced constrained access to contraception, with women reporting delayed and inaccessible reproductive health care based upon research in the early months of the pandemic [10]. Shifting policies during COVID-19 and need for health protection against the virus may contribute to greater reliance upon the internet as a source of abortion information and services, ranging from those fully supported by health care providers to fully self-managed abortions [11].

**Figure 1:**
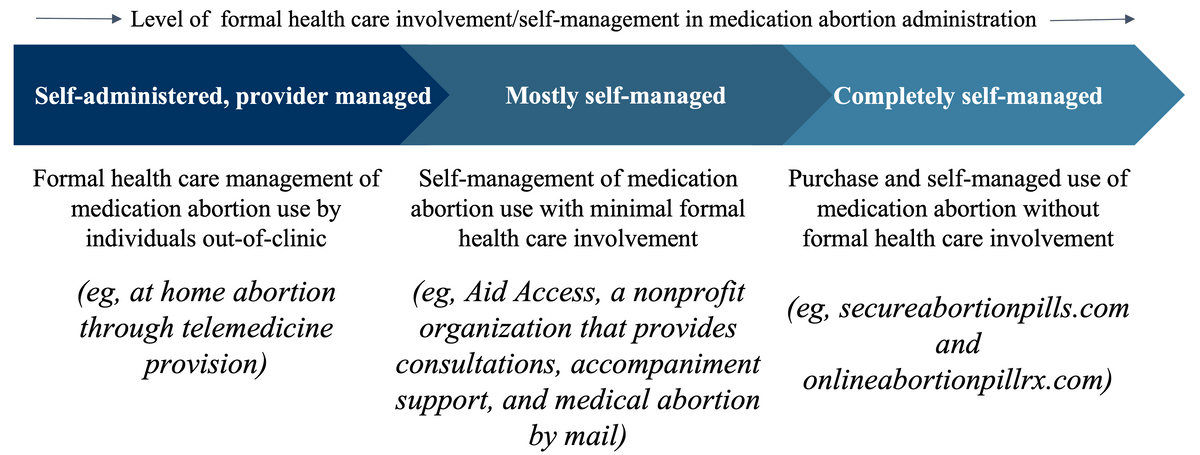
Spectrum of out-of-clinic medication abortion management.

Medication abortion via pills administered at 10 weeks’ gestation or less is considered a safe and effective method for pregnancy termination, both in clinics with provider supervision and when delivered remotely via telemedicine [12,13]. Evidence suggests that many women choose home abortions for privacy, affordability, and convenience [2,13-15]. Others may go outside of the traditional US health care system to get abortions through entirely self-managed abortion [14] or web-based medication abortion services that offer remote provider support [15]. There appears to be a spectrum of formal health care system involvement and self-management for these out-of-clinic abortions, which we outline in a conceptual framework (Figure 1) informed by recent research [16,17].

The majority of adults in the United States use the internet to look for health information, most often via Google [18,19]. Web-based searches present a valuable data source for examining health-seeking behaviors and concerns at a population level and the quality of information on websites, thereby advancing the field of infodemiology. Infodemiology is the science of distribution and determinants of information on the internet or in a population, with the aim to inform public health and public policy [20]. We analyzed Google searches to understand health concerns and impactful sources of online health information on out-of-clinic abortions. A study conducted in 2017 showed that there is interest to learn more about “self-abortion” on Google, especially among adolescents and young women facing an unintended pregnancy [21]. We built on recent research using Google Trends that found a high volume of information seeking for *abortion pill* and for *abortion* and wide variations by state policies in the United States in 2018 [22]. Notably, across states a higher volume of abortion pill searches was associated with more concerns about access to contraceptives, higher unplanned pregnancy rates, and fewer abortion facilities. Given the proliferation of telemedicine abortion during the pandemic and an increase in self-managed abortions [23,24], we sought to answer 3 research questions:

1. During 2020, to what extent did people search for out-of-clinic abortions and when did search interest peak? Did search trends differ in the first quarter (before the pandemic was officially declared by the World Health Organization [WHO] on March 11) compared with the rest of the year when the pandemic surged?
2. When users searched for key initial search terms, what other search queries were users most often also searching for and which queries had the highest relative search volume (RSV)?
3. How did the relative popularity of the initial search terms vary across states?

## Methods

Guided by a literature review [9,10,14-17,21-26] and using an iterative process, we retrieved Google Trends query data on the keywords *home abortion, self abortion,* and *buy abortion pill online* as search terms. Each search term was found to be related to the topic *abortion*, while *home abortion* and *buy abortion pill online* were also related to the topic *medical abortion*. Abortion and abortion pill as keyword searches on Google have been shown to correlate with unwanted pregnancies, concerns for contraceptive access, and lack of abortion care facilities [22]. As *home abortion, self abortion,* and *buy abortion pill online* contained more than 1 word, we tested these keywords using double quotation marks; however, upon retrieval search data were unavailable or of insufficient quality. We also tested *self-abortion* (with a dash) and ran into similar limitations. While *home abortion* is a broad search term that we saw as encompassing both provider and self-managed abortion, we also explored if the Google Trends website returned results for narrower keywords including *home abortion through telemedicine*, *self induced abortion,* and *self managed abortion,* but search data for these were unavailable.

We followed the core procedures of the simulation protocol described by Zepecki et al [25] and Mavragani and Ochoa [26] where appropriate. To answer the first research question, we retrieved Google Trends data using the Explore function for the period from January 1, 2020, to January 1, 2021, in the United States. This timeframe allowed us to plot weekly data and assess trends and search peaks for the selected keywords. We also compared trends before COVID-19 was declared a pandemic (week including January 1 through week of March 1-7, 2020) with trends observed during the pandemic (week of March 8-14 through week of December 27, 2020-January 2, 2021). We selected the “health” category and the “website” category. Subcategories were not selected when searching for keywords. We examined the relative search index (RSI) for each of our initial search terms. The RSI values reflect the normalized popularity of each initial search term relative to all other Google searches in a given geolocation (in our case, the United States) for January 1, 2020, to January 1, 2021. As search data are normalized and indexed 0-100, where 100 denotes the maximum search interest for a given search term in the time and location selected and 0 denotes no interest, the RSI values for each of the initial search terms selected inform us which terms are relatively more popular as a proportion of all searches on all topics on Google at the chosen time and geographic location. RSI values also show changes in relative popularity over time, allowing us to identify peak interest times. For instance, if we examine the keyword *abortion pill* within a single year in the United States, we might find that it has an RSI of 95 in January and then declines as months go by in that year. This would suggest that the peak interest in that term within the United States was at the beginning of that year.

To answer the second research question, we first used the Google Trends application programming interface (API) to access data to ascertain the top search queries associated with each of our initial search terms and their respective RSI values. Subsequently, we used the Google Health Trends API to ascertain the normalized proportion of searches for a specific query out of the sum of searches for a set of top queries associated with the initial search term. This proportion is known as the RSV [25]. To address the third question, we used the Google Trends website to explore the state-specific RSI values for each of our initial search terms, reflecting normalized regional popularity of each search term compared with other states within the United States for the designated period.

The RSI and RSV values returned by the Google APIs are based on a daily cached random sample of the universe of all Google search data in the specified geolocation. Consequently, any queries with low search volume can sometimes return “no data” even if a new random sample returns valid data. These events produce fluctuations in the top queries retrieved with the Google Trends and Health Trends APIs across samples [27]. Following an approach used by Pew Research Center scholars [28], we adapted our methodology to create nonmissing average measures for RSI and RSV values, calculated from resampled results for each initial search term. Specifically, from the Google Trends webpage, we pulled 30 unique data samples for *home abortion*, *self abortion,* and *buy abortion pill online* and estimated the nonmissing average RSI over time and by state. We used a similar resampling approach to compile data from the Google Trends API and Health Trends API. To alleviate concerns about idiosyncratic data extractions on a given date, half of the 30 samples were pulled between April 1 and 21 and half between June 4 and 18, 2021.

Finally, to help contextualize the search interest in out-of-clinic abortions, we used the Custom Search API [25]. This API allowed us to obtain the top 10 webpages linked to each of the 3 initial search terms determined by Google’s search engine optimization algorithm as of April 4, 2021. A previous study suggested that these webpages receive at least 92% of the search traffic [29]. Webpage probabilities, or the likelihood that a user would click-through that webpage search result on Google, were assigned based on research by Chitika Insights [29].

## Results

### Search Traffic Over Time and Top Queries

**Figure 2:**
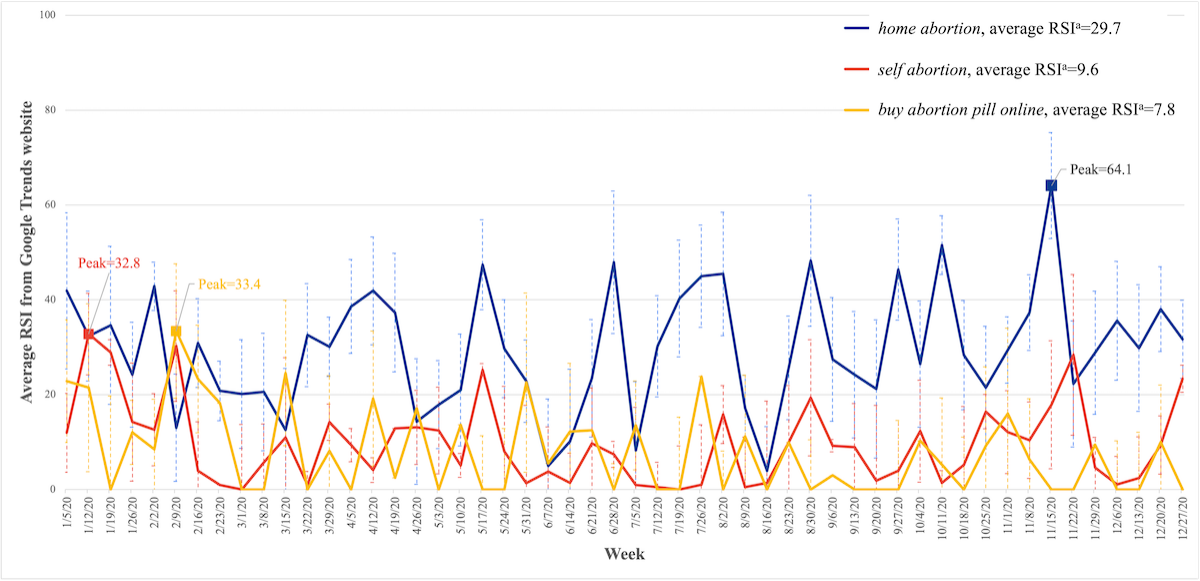
Nonmissing mean RSI (relative search index) values for *home abortion*, *self abortion*, and *buy abortion pill online* for January 1, 2020, to January 1, 2021. ^a^Mean RSI calculated based on 30 unique data samples for January 01, 2020, to January 01, 2021, pulled from the Google Trends website for "health" queries only between April 01, 2021, and June 18, 2021. Nonmissing means presented with associated standard deviations.

Figure 2 compares the average RSI values over time for *home abortion, self abortion*, and *buy abortion pill online*. As shown, *home abortion* was the most popular of the 3 initial search terms explored. *Home abortion* had an average popularity search index relative to all other searches in the United States (RSI) of 30 in 2020 compared with almost 10 for *self abortion* and 8 for *buy abortion pill online*. Searches for *home abortion* remained higher throughout the year compared with those for the 2 other search terms we explored, and peaked in November. By contrast, searches for *self abortion* and *buy abortion pill online* peaked in January and February of 2020, prior to the official onset of the COVID-19 pandemic, although there was less variability in these searches over time and these peaks do not reflect significantly greater relative interest compared with other weeks in 2020.

**Figure 3:**
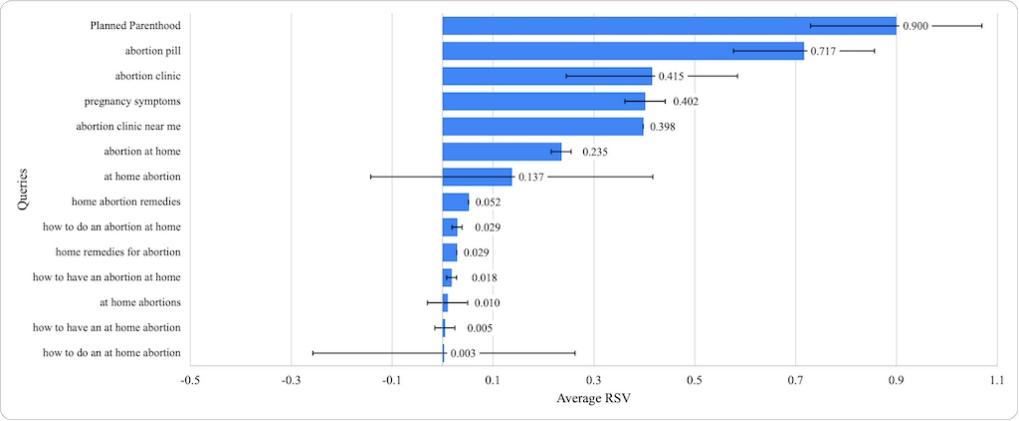
Nonmissing mean RSVs (relative search volumes) for top ranked queries associated with *home abortion* in the United States for January 1, 2020, to January 1, 2021. Nonmissing averages presented with associated standard deviations.

There were many top ranked queries associated with *home abortion,* including *abortion at home*, *at home abortion(s)*, *abortion pill*, *abortion remedies*, *abortion clinic*, *home remedies for abortion*, *pregnancy symptoms*, *how to have an at home abortion*, *how to do an at home abortion*, *how to do an abortion at home*, *Planned Parenthood*, and *abortion clinic near me*. Relative to all these top queries, *Planned Parenthood* had the highest RSV, followed by *abortion pill* and *abortion clinic* (Figure 3). In comparison, specific queries on *abortion at home*, *home abortion remedies*, and *how to do an abortion at home* or *how to have an abortion at home* were popular but had lower search volumes.

*Self induced abortion* was the only consistent top query for *self abortion*. Similarly, *buy abortion pill online* was associated with only 1 query, *buy abortion kit online*.

### Search Traffic Across States

As shown in Table 1, among the 10 states with highest average RSI for *home abortion,* the majority were those where abortion was severely restricted (5/10) or restricted (3/10). Except for Pennsylvania, none of the top states with abortion restrictions were located in the Northeast. Conversely, New York and Connecticut, 2 states in the Northeast where abortion was accessible, also topped the list with highest searches for *home abortion*. Similarly, the majority of states with high average RSI for *self abortion* were severely restricted (2/10) or restricted (4/10) and some states where abortion is accessible, such as Massachusetts, New Jersey, Vermont, and Illinois, also topped the list for *self abortion* searches. There was no overlap in high-search states for these 2 initial search queries, suggesting differences in state-level interest in *home abortion* and *self abortion* searches. Google did not provide results for top states with the highest RSI for *buy abortion pill online*.

**Table 1:** Top 10 US states with the highest search traffic for home abortion and self abortion in 2020.

Rank	*Home abortion*				*Self abortion*		
	State	Average RSI^a^ (SD)	Abortion access	State		Average RSI (SD)	Abortion access
1	Indiana	0.7 (0.33)	Severely restricted	Alabama		0.61 (0.44)	Severely restricted
2	Arizona	0.69 (0.33)	Restricted	Virginia		0.5 (0.38)	Restricted
3	New York	0.65 (0.35)	Accessible	Florida		0.4 (0.25)	Restricted
4	Pennsylvania	0.65 (0.36)	Restricted	Massachusetts		0.39 (0.33)	Accessible
5	Connecticut	0.64 (0.38)	Accessible	Georgia		0.30 (0.25)	Severely restricted
6	Missouri	0.63 (0.41)	Severely restricted	New Jersey		0.29 (0.33)	Accessible
7	Iowa	0.62 (0.42)	Restricted	North Carolina		0.26 (0.29)	Restricted
8	Kentucky	0.62 (0.37)	Severely restricted	Illinois		0.23 (0.21)	Accessible
9	Ohio	0.62 (0.29)	Severely restricted	Pennsylvania		0.22 (0.26)	Restricted
10	Arkansas	0.61 (0.39)	Severely restricted	Vermont		0.20 (0.42)	Accessible

^a^RSI: relative search index.

### Top Webpages for *home abortion, self abortion,* and *buy abortion pill online* Searches

To better provide contextual evidence for our results, we took a snapshot of the top 10 webpages presented to users searching for *home abortion, self abortion,* and *buy abortion pill online* in the United States as of April 2021 (Multimedia Appendix 1). Regarding *home abortion*, only 2 of the top ranked webpages focused on health education, 1 from Healthline, a health blog that cautions against abortion home remedies while recommending that women consider physician-prescribed medication abortion at home; the other, an antiabortion blog from a Wisconsin clinic that focuses on the abortion process and on ways of reversing medication abortion. These 2 sites ranked first and ninth, respectively. A Wikipedia overview article on self-induced abortion ranked eighth. Several webpages focused on potential access expansions to home abortions under the Biden administration and to mail-order abortion pills approved in the United Kingdom during the pandemic. Webpages covering news and scholarly articles were more common than webpages from clinical settings.

For *self abortion,* webpages from the Guttmacher Institute (a proabortion advocacy organization) ranked first and third, followed by several academic publications.

As many as 4 out of 10 top webpages for *buy abortion pill online* were sponsored by prochoice groups such as Planned Parenthood, Vox, Ms. Magazine, and Plan C; these webpages openly discuss where and how to get abortion pills online. In contrast, 4/10 webpages presented antiabortion content, 2 managed by Crisis Pregnancy Centers that dissuade people from buying the abortion pill; 1 from a Florida county promoting a referendum to declare itself a “Pro-Life Sanctuary”; and 1 from a business blog cautioning potential online pill buyers from ending in jail because they are doing an illegal activity. Notably, the top webpage from the National Pharmaceutical Provider Association does not include any content on medication abortion. Images of top webpages for *buy abortion pill online* that were links to internal website search results providing no relevant content for medication abortion are presented in Multimedia Appendix 2 (webpages ranked 1, 7, and 10). The search query used for each of these internal searches included emojis or special characters or both.

## Discussion

### Principal Findings and Comparison With Prior Work

The use of medication abortion has been increasing steadily in the United States since its introduction in 2000, accounting for just 6% of all induced abortions in 2001 and almost 40% in 2017 [12]. Such steady growth in uptake of medication abortion coupled with increased access barriers to abortion, including recent disruptions related to COVID-19, may have accelerated the use of out-of-clinic-abortion services in the United States in 2020 [23,24]. We aimed to describe, in near–real-time, population interest in out-of-clinic abortion information and services during the pandemic on Google.

In 2020, *home abortion* was the most popular search term among those we explored. As shown by the multiple associated queries, this search term reflects interest from searchers in out-of-clinic abortion care. We cannot know the precise type of out-of-clinic abortion information or services being sought by consumers searching *home abortion.* However, we do know that there were more searches for *home abortion*—which could encompass any abortion happening at home regardless of clinical support—than for *self abortion,* a term that we believe implies an interest in self-management of abortion, and *buy abortion pill online*, a term that implies an interest in self-procurement of medication abortion.

Of the multiple top queries for *home abortion*, *Planned Parenthood* received the highest search traffic relative to other search queries for this keyword (based on RSV), followed by *abortion pill*. The higher search traffic may be associated with the high ranking of the Planned Parenthood website on Google. A previous study indicated that Planned Parenthood was the top webpage for medication abortion searches on Google and the site that provides the most accurate information on medication abortion [30]. Additionally, the higher RSV for Planned Parenthood in relation to *home abortion* searches may reflect an interest in out-of-clinic abortion involving a provider that can give oversight, medications, and support for an abortion at home and the role of Planned Parenthood as a recognized abortion provider offering telemedicine abortion services both prior to and during the pandemic [31]. In this model, clinicians remotely prescribe medication abortions by collaborating with Planned Parenthood centers that do not have on-site abortion providers. The patient visits their laboratories, undergoes ultrasound, and receives medications from their local Planned Parenthood Clinic.

Until 2020, the US Food and Drug Administration (US FDA) required certified providers to dispense mifepristone, 1 of the 2 drugs in the abortion regimen most commonly prescribed, in clinics or hospitals [9]. However, a federal district court ruled in July 2020 that the US FDA was required to lift this restriction and allow remote distribution of mifepristone via telemedicine during the pandemic. We note that searches for *home abortion* peaked in November of 2020, during the third wave of the COVID-19 pandemic and national allowance of telemedicine provision of medication abortion [8,9].

Novel platforms such as AidAccess and the Plan C Campaign are facilitating the online provision of abortion pills by offering information, support for, and access to medications for self-managed abortion [9,24]. Our results for *self abortion* and *buy abortion pill online*, searches that denote minimal provider support or fully online self-managed abortion care, showed that searches for *self abortion* were slightly more popular, but data for both were sparse. Our findings indicate relatively low population interest in these search queries for Google searchers in general, and compared with *home abortion*. Minimally supported or totally self-managed medication abortion with the 2-drug regimen of mifepristone and misoprostol is difficult to implement in the United States, even in states considered supportive of abortion rights, because ordering the drugs online through direct order without a prescription is considered illegal and many foreign clinics or pharmacies do not ship to the United States. By contrast, clinically supported home abortions may overcome multiple legal, economic, and cultural barriers because they may be more private, convenient, affordable, and less stigmatizing than in-clinic abortions. Aiken et al [24], using AidAccess data spanning January 2019 through April 2020, found an increase in the rate of requests for self-managed medication abortion in the United States [24]. Nonetheless, past research estimated that 7% of women in the United States would attempt to self-manage an abortion during their lifetime [14]. Notably, we found that searches for these 2 queries in 2020 peaked before the pandemic, in contrast to searches for home abortion that peaked during a third wave of COVID-19, during a time when telemedicine provision of medication abortion was allowed.

Previous studies have shown that legal restrictions to abortion do not reduce desire or intention to seek abortion care, but may push abortion seekers to virtual sources of information and services [15,24]. In light of such findings, our study provides important contextual evidence about the differences in relative queries across states with varying social attitudes and legal positions on abortion. *Home abortion* searches were predominantly most popular in states with restricted abortion access such as Arizona, Missouri, Arkansas, Indiana, and Kentucky. Arizona, Missouri, and Arkansas are 3 of the 5 states that prohibit the use of telemedicine for abortion while Indiana prohibits prescription of medication abortions without a prior in-person patient examination [9,31]. In 2020, Kentucky and Arizona enacted laws requiring physical presence of prescribing clinicians, hence effectively blocking the use of telemedicine [9]. Furthermore, several of the states with highest search traffic for *home abortion* attempted to limit abortion access during the COVID-19 outbreak by deeming abortion “non-essential” [32].

We saw the highest search traffic for *self abortion* among different states than for *home abortion*. Although *self abortion* traffic was mostly concentrated in Southern states where abortion is more restricted, traffic also came from 4 states, predominantly located in the Northeast, that make abortion accessible via telemedicine and by allowing nonphysician-certified clinicians to authorize the medication abortion [33]. Previous research suggested that barriers to clinic access are present even in states with more supportive abortion policies [15]. In these states, barriers to abortion include cost, difficulties taking time away from work or arranging childcare, abortion stigma, and the need to keep an abortion secret for fear of negative consequences [15].

As people turn to the internet for information and resources on out-of-clinic abortion, they can face challenges to informed reproductive choice and abortion access. Consistent with previous research on abortion webpages [30], we found that the top webpages listed in our snapshots provided limited evidence-based clinical content on self-management of abortions, particularly abortions without clinical provider supervision. The webpages linked to *buy abortion pills online* were neither relevant nor helpful. In fact, some pages offered no content related to abortion (self-managed or otherwise) and their appearance as top search results could be related to efforts to leverage search algorithm optimizations to appear higher on search results either through spam or erroneous linking. Furthermore, several sites presented disinformation about abortion, the pill, and other aspects of sexual and reproductive health, a finding that aligns with past research on the contents of abortion search results on Google [30,34,35]. Multiple legal, financial, cultural, and logistical barriers to abortion care underscore the need to support consumer access to accurate webpages that provide high-quality information and resources.

### Limitations

Our study faced several limitations. Although we researched 3 keywords for out-of-clinic abortions and their associated top queries, it is not an exhaustive list of every term searched. For instance, we considered *telemedicine abortion* and *telehealth abortion*, but Google did not give results for these search queries. Moreover, we were not able to identify the number of unique users or their individual characteristics, nor the reasons that prompt individuals to search for out-of-clinic abortion. Additionally, this research did not compare searches in 2020 for out-of-clinic abortion terms with previous years. Rather, we chose to scope our study to pre- and post-pandemic US searches within 2020. Nonetheless, additional sensitivity analyses comparing searches in 2020 with those in 2018 and 2019 showed similar search trends for *home abortion* and *self abortion.* Further research should be done to exhaustively explore differences in searches over time, in consideration of the impacts of changes in the volume of all Google searches over time on the frequency of searches for abortion-related terms. For example, time-series heatmaps and other visual representations of key terms by geographic region could be useful in future research and as general tools for understanding these trends.

We also cannot assume that online searches for out-of-clinic abortion reflect intention to use or current use of this type of abortion care. Google data allow us to assess relative population-level search interest and concerns about key topics and search queries in near or real-time. However, Google Trends only shows data for popular queries, so search queries with low volume appear as “0.” The Google Health Trends API does not give RSV below a certain threshold (unknown to us). Following prior research, we addressed this limitation by implementing a resampling approach over several months. We treated each of 30 data extractions from the Google Trends and Health Trends API as an independent sample and calculated average measures (RSI score and RSV score with nonmissing averages). We believe this is a valid way to account for inherent sampling fluctuations created by Google’s own mechanisms that intentionally obfuscate precise search activity at any one point in time [36]. We urge other researchers to consider the resampling approach in future analyses of infrequent Google search data.

Additionally, although we chose our initial search queries carefully, with consideration of relevant literature and search interest, further research should explore other search queries related to self-management of abortion to establish user interest for other relevant queries. As for top webpages that searchers of out-of-clinic abortion are shown on Google, we took a snapshot of these based on a 1-day retrieval of data; these listings and rankings are likely to fluctuate over time. Future research should examine top webpages and their rankings by frequent resampling over time and do a thorough content analysis to gain further insights into the content and quality of webpages providing information on abortion self-management to people searching on Google.

### Conclusions

Our analysis provided meaningful insights into population-level interest in out-of-clinic medication abortions in the United States during the first year of the pandemic. Our findings demonstrate greater interest in home abortions, which presumably have varying degrees of clinical support than in minimally or nonclinically supported self-induced abortions. While our study was mainly descriptive, showing ways in which infrequent abortion-related search data can be analyzed, future studies should explore correlations between the keywords denoting interest in out-of-clinic abortion and abortion care measures and test models that allow for improved monitoring and surveillance of abortion concerns in our rapidly evolving policy context.

## Appendix

Multimedia Appendix 1 A snapshot of top webpage results for *home abortion*, *self abortion*, and *buy abortion pill online* searches in the United States as of April 4, 2021.

Multimedia Appendix 2 Images of top webpages for *buy abortion pill kit online* presenting internal site search results.

## Abbreviations

API: application programming interface
US FDA: US Food and Drug Administration
RSI: relative search index
RSV: relative search volume
WHO: World Health Organization
