# Supplementary material for: Exploring Google Searches for Out-of-Clinic Medication Abortion in the United States During 2020: Infodemiology Approach Using Multiple Samples
Source: JMIR Infodemiology. 2022 May 12;2(1):e33184. doi: 10.2196/33184 (PMC10014087; doi:10.2196/33184)
Supplement: Multimedia Appendix 1 [file infodemiology_v2i1e33184_app1.docx]

**Appendix 1. A snapshot of top webpage results for 'home abortion' searches in the US as of April 4, 2021**

Table 1. A snapshot of top webpage results for 'home abortion' searches in the US as of April 4, 2021

| **Webpage rank** | **Webpage probability** | **Webpage url** | **Webpage type** | **Webpage summary** |
| --- | --- | --- | --- | --- |
| 1 | 0.325 | <https://www.healthline.com/health/abortion-home-remedy> | Health education page | Health blog post on Healthline (health information blog) called “Abortion Home Remedies Aren’t Worth the Risk, But You Still Have Options”. Includes link to Aid Access and references other legal, financial resources. |
| 2 | 0.176 | <https://www.politico.com/news/2021/03/20/abortion-pills-telemedicine-477234> | News article | News article on Politico (political North American news) called “Will at-home abortions make Roe v. Wade obsolete” published March 21, 2021. |
| 3 | 0.114 | <https://www.bbc.com/news/newsbeat-52092131> | News article | News article on BBC (public service news source based in the UK) called “Coronavirus: Home abortions approved during outbreak”, focused on home abortions in the UK (published March 31, 2020). |
| 4 | 0.081 | <https://www.bpas.org/abortion-care/abortion-treatments/the-abortion-pill/remote-treatment/> | Health services page | Webpage on the site for the “British Pregnancy Advisory Service” (UK abortion care service) called “Pills by Post – Abortion Pill treatment at home” providing information on steps involved in accessing mail-order medication abortions for women in England and Wales, including risks of abortion pills and contents of treatment package. |
| 5 | 0.061 | <https://pubmed.ncbi.nlm.nih.gov/21556304/> | Academic publication in Bulletin of WHO | Scholarly research article called “Comparative effectiveness, safety and acceptability of medical abortion at home and in a clinic: a systematic review” (published May 1, 2011, systematic review article by articles based in the UK). |
| 6 | 0.044 | <https://www.whitehouse.gov/briefing-room/presidential-actions/2021/01/28/memorandum-on-protecting-womens-health-at-home-and-abroad/> | Government press release | Official statement from the White House Briefing room called “Memorandum on Protecting Women’s Health at Home and Abroad” revoking the Mexico City Policy that withheld family planning funds from agencies that perform abortion (28 January 2021). |
| 7 | 0.035 | <https://pubmed.ncbi.nlm.nih.gov/22913927/> | Academic publication | Scholarly qualitative research article called “Autonomy and dependence--experiences of home abortion, contraception and prevention” (published, Septemberr 27, 2013, in Scandinavia J Caring Science). |
| 8 | 0.031 | <https://en.wikipedia.org/wiki/Self-induced-abortion> | Non-profit (open source information page) | Wikipedia article on “Self-induced abortion” with discussion of methods, rates, history, and law. |
| 9 | 0.026 | <https://vidamedicalclinic.org/at-home-abortion-info/> | Health education page | Webpage on anti-abortion webpage for “Vida Medical clinic” with content on at-home abortion process, side-effects, pregnancy testing resources, and “reversing” an at-home abortion. |
| 10 | 0.024 | <https://www.vox.com/the-highlight/2019/7/1/18638649/abortion-pill-internet-misoprostol-mifepristone> | News article | New article on Vox (popular news source) called “A boom in at-home abortions is coming” (published on July 9, 2019) documenting anecdotal evidence that self-managed abortions are on the rise. |

Table 2. A snapshot of top webpage results for 'self-abortion' searches in the US as of April 4 2021

| **Webpage rank** | **Webpage probability** | **Webpage url** | **Webpage type** | **Webpage summary** |
| --- | --- | --- | --- | --- |
| 1 | 0.325 | <https://www.guttmacher.org/article/2019/11/self-managed-abortion-may-be-rise-probably-not-significant-driver-overall-decline> | Non-profit (open source research/information page) | Blog post from Guttmacher called “Self-Managed Abortion May Be On The Rise, But Probably Not A Significant Driver Of The Overall Decline In Abortion” describing self-managed abortion in the US and how it is measured (published July 11, 2019). |
| 2 | 0.176 | <https://www.npr.org/2019/09/19/759761114/with-abortion-restrictions-on-the-rise-some-women-induce-their-own> | News article | News article on NPR (independent, non-profit media) “With Abortion Restrictions On The Rise, Some Women Induce Their Own”, mentions Aid Access and links out to Plan C (published September 19, 2019) . |
| 3 | 0.114 | <https://www.guttmacher.org/gpr/2018/10/self-managed-medication-abortion-expanding-available-options-us-abortion-care> | Academic publication | Guttmacher Policy Review article called “Self-Managed Medication Abortion: Expanding the Available Options for U.S. Abortion Care” (published October 17, 2018). |
| 4 | 0.081 | <https://pubmed.ncbi.nlm.nih.gov/31859163/> | Academic publication | Scholarly research article called “Self-managed abortion: A systematic scoping review” (published in 2020). |
| 5 | 0.061 | <https://en.wikipedia.org/wiki/Self-induced_abortion> | Non-profit (open source information page) | Wikipedia article on “Self-induced abortion” with discussion of methods, rates, history, and law. |
| 6 | 0.044 | <https://pubmed.ncbi.nlm.nih.gov/32150279/> | Academic publication | Scholarly research article called “Self-administered versus provider-administered medical abortion” presenting a systematic Cochrane review comparing the effectiveness, safety, and acceptability of self-administered versus provider-administered medical abortion in any setting (published in 2020). |
| 7 | 0.035 | <https://jamanetwork.com/journals/jamanetworkopen/fullarticle/2774320> | Academic publication | Scholarly research article called “Prevalence of Self-Managed Abortion Among Women of Reproductive Age in the United States” presenting results of a cross-sectional survey estimating the prevalence of self-managed abortion in the US from ANSIRH (published in 2020. |
| 8 | 0.031 | <https://www.ipas.org/our-work/abortion-self-care/> | Health education page | Information webpage on Ipas website (reproductive health access and rights programs and research organization) describing their stance of ‘self care abortion’, providing information on doing it safely with links to other pages on their sire, and descriptions of their current projects supporting/promoting self-managed abortion care and training both in the US and globally. |
| 9 | 0.026 | <https://www.healthline.com/health/abortion-home-remedy> | Health education page | Health blog post on Healthline (health information blog) called “Abortion Home Remedies Aren’t Worth the Risk, But You Still Have Options”. Includes link out to Aid Access. |
| 10 | 0.024 | <https://bmcwomenshealth.biomedcentral.com/articles/10.1186/s12905-019-0877-0> | Academic publication | Scholarly research article called “Texas women’s decisions and experiences regarding self-managed abortion” reporting findings of surveys and interviews with women in Texas on motivations for and experiences with attempts to self-manage an abortion (published in 2020). |

Table 3. A snapshot of top webpage results for 'buy abortion pill online' searches in the US as of April 2021

| **Webpage rank** | **Webpage probability** | **Webpage url** | **Webpage type** | **Webpage summary** |
| --- | --- | --- | --- | --- |
| 1 | 0.325 | <https://www.pcmanet.org/page/2/?s=%F0%9F%93%BA%F0%9F%8D%99+Buy+Abortion+Pill+Online+%3A+%F0%9F%8C%BF+www.OrderAbortionPill.com+%F0%9F%8C%BF+-+Cheapest+Abortion+pills+Without+Prescription> | National pharmaceutical provider network page | Search results on PCMA website (Pharmaceutical Care Management Association), results presented do not include anything on medication abortion specifically. However, they do offer members a web-based platform to communicate and collaborate. Members have taken on abortion-related issues such as whether to boycott meetings in LA and TX that have passed heartbeat bills. |
| 2 | 0.176 | <https://www.plannedparenthood.org/learn/abortion/the-abortion-pill/how-do-i-get-the-abortion-pill> | Health education and services page | Webpage on Planned Parenthood webpage called “How do I get the abortion pill?”, subpage on the abortion pill information page that includes content on how to get the abortion pill, using telemedicine, cost, and information about participating in TelAbortion study. |
| 3 | 0.114 | <https://www.vox.com/science-and-health/2018/10/20/17999996/abortion-mail-online-mifepristone-misoprostol> | News article | News article on Vox (popular news source) called “Abortions by mail are available now in the US. Here’s what you need to know.” describing and linking out to Aid Access and mail-order abortion legality in the US (published October 22, 2018). |
| 4 | 0.081 | <https://msmagazine.com/2020/09/30/honeybee-health-us-based-online-pharmacy-first-to-ship-abortion-pills-to-patients-inside-the-u-s/> | News article | News article on Ms. Magazine (women’s rights magazine started in the 1970s) website called “U.S.-Based Online Pharmacy First to Ship Abortion Pills to Patients Inside the U.S.” focused on legal provision of medical abortion by mail in 2020 by Honeybee Health including reference to Plan C (published September 30, 2020). |
| 5 | 0.061 | <https://www.plancpills.org/> | Health education page | Home webpage on Plan C website, which provides abortion information and resources (including links out to various at-home medication abortion providers). |
| 6 | 0.044 | <https://northcareclinic.org/is-it-safe-to-purchase-the-abortion-pill-online/> | Health education and services page | Webpage called “Is it safe to purchase the abortion pill online” on an anti-abortion (CPC) website. CPC located in Pennsylvania. Webpage includes seven reasons not to order the abortion pill online, listing that the FDA requires physician oversight, it could be illegal, the contents are questionable, abortion pill is a medical procedure, abortion pill cost is too high, you deserve professional care—all pushing people to contact their clinic before seeking an abortion. |
| 7 | 0.035 | <https://srctc.com/search/%E2%A0%A5%E2%A1%BF+Buy+Abortion+Pill+Over+The+Counter+-+%E2%AD%90+www.OrderAbortionPill.com+%E2%AD%90+Pills+without+prescription+%E2%A1%BF%E2%A0%A5Buy+Abortion+Pill+Online+Without+Prescrpiton/> | Internal website search results, county services page (tax collection) | Search results on Santa Rosa County Florida, Tax Collector website for “Buy Abortion Pill Over The Counter www.OrderAbortionPill.com Pills without prescription ⡿⠥Buy Abortion Pill Online Without Prescription”. Provides no direct search results. However, County residents voted on November 3 2020 to declare the county a “Pro-Life Sanctuary”. |
| 8 | 0.031 | <https://www.fastcompany.com/90468030/how-an-online-search-for-abortion-pills-landed-this-woman-in-jail> | News article | News article on Fast Company (business media brand) called “How an online search for abortion pills landed this woman in jail” presenting the case of Latice Fisher (and others) with discussion of the risks of search histories and online footprints for people who have sought medication abortion online. |
| 9 | 0.026 | <https://prcgr.org/can-i-buy-abortion-pills-online/> | Health education and services page | Webpage called “Can I buy abortion pills online?” on an anti-abortion (CPC) website. Discusses abortion protocol before COVID-19, safety concerns, meet with your doctor, and “don’t forget to breathe” (abortion delay tactic), all seeking to get people to seek care with them before seeking an abortion. |
| 10 | 0.024 | <https://nvbar.org/?s=Buy%20Abortion+Pill+Online%20%F0%9F%8D%BB%F0%9F%92%89%E2%9B%B8%20Abortion+Pills%20without%20prescription%20on%20%F0%9F%91%A9%E2%80%8D%E2%9A%95%EF%B8%8F%20www.OrderAbortionPill.com%20%F0%9F%91%A9%E2%80%8D%E2%9A%95%EF%B8%8F%20-%20Where%20Can%20You%20Get%20Abortion+Pills%20%E2%9B%B8%F0%9F%92%89%F0%9F%8D%BBOnline> | Internal website search results, state Bar association | Search results on State Bar of Nevada website for “Buy abortion pill online …”. No search results found on this site. |
