# Supplementary material for: Exploring Google Searches for Out-of-Clinic Medication Abortion in the United States During 2020: Infodemiology Approach Using Multiple Samples
Source: JMIR Infodemiology. 2022 May 12;2(1):e33184. doi: 10.2196/33184 (PMC10014087; doi:10.2196/33184)

**Appendix 2. Images of top webpages for ‘buy abortion pill kit online’ presenting internal site search results**

Image 1: Webpage 1, internal search results on Pharmaceutical Care Management Association website


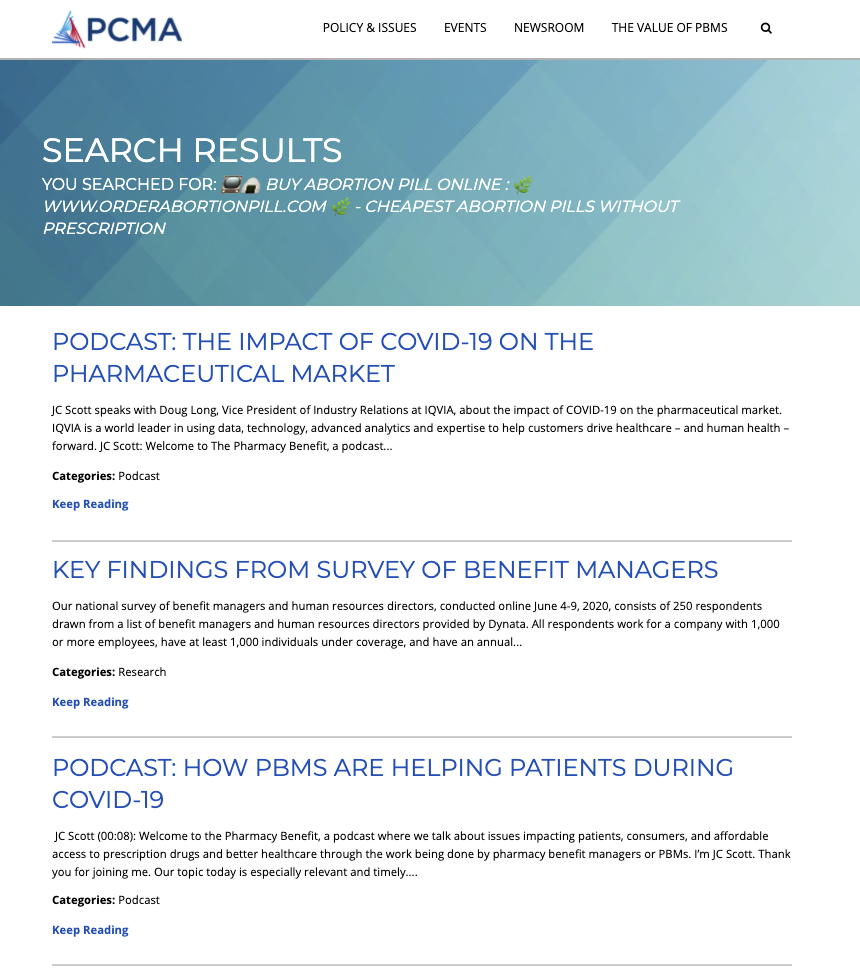


Image 2: Webpage 7, internal search results on Santa Rosa County Tax Collector website


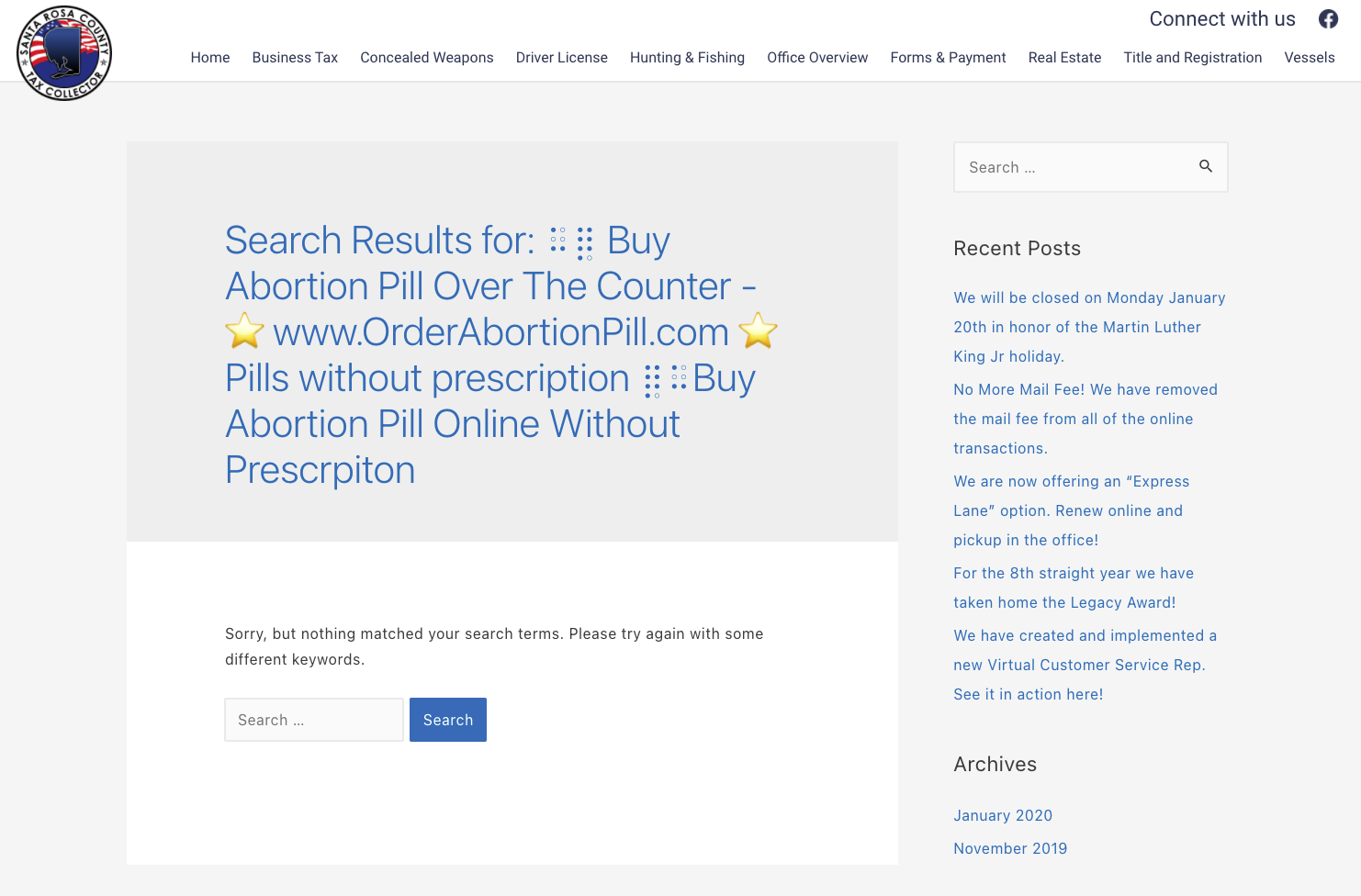


Image 3: Webpage 10, internal search results on State Bar of Nevada website


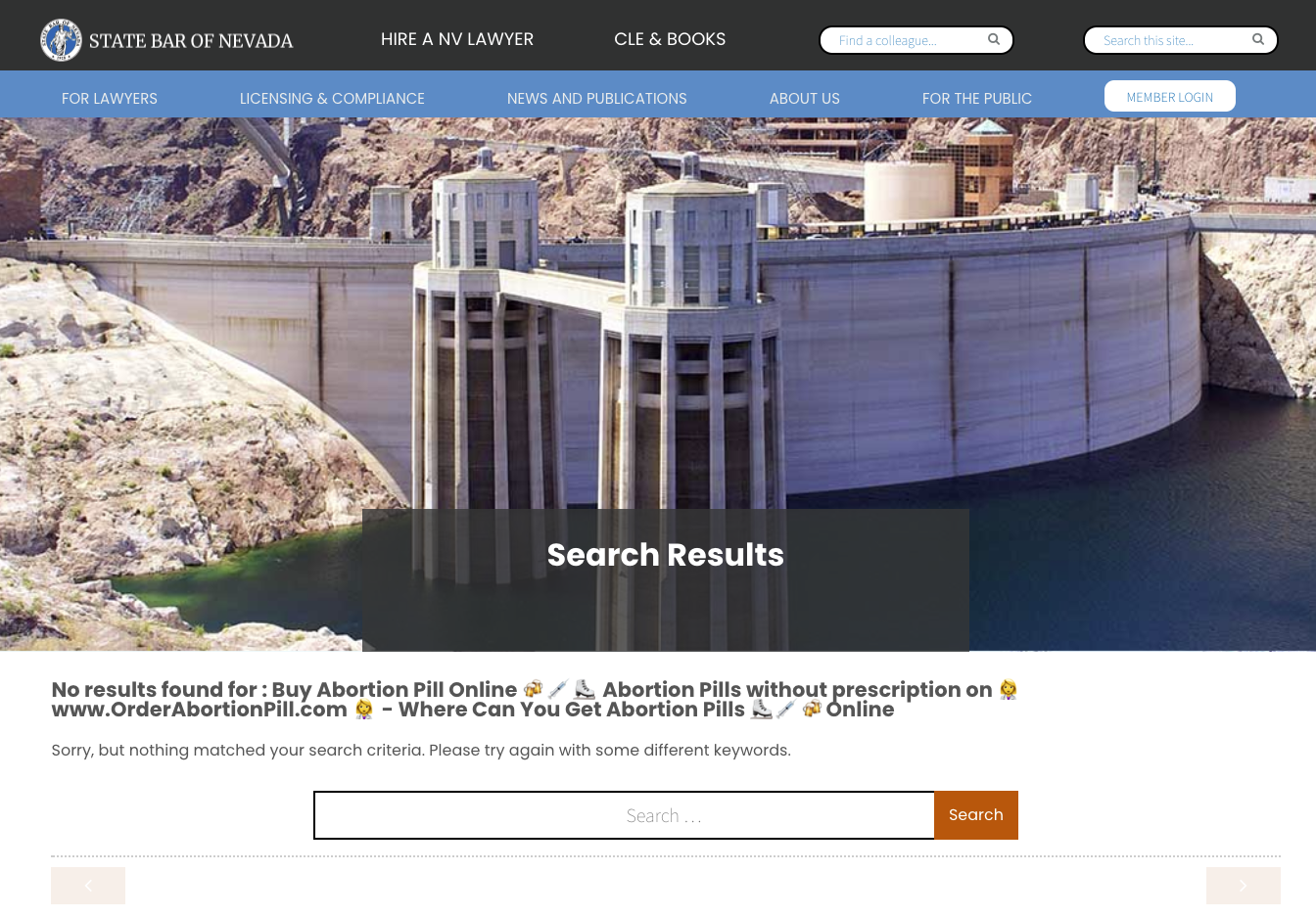

Supplement: Multimedia Appendix 2 [file infodemiology_v2i1e33184_app2.docx]
